# Tracking a TGF-β activator in vivo: sensitive PET imaging of αvβ8-integrin with the Ga-68-labeled cyclic RGD octapeptide trimer Ga-68-Triveoctin

**DOI:** 10.1186/s13550-020-00706-1

**Published:** 2020-10-31

**Authors:** Neil Gerard Quigley, Katja Steiger, Frauke Richter, Wilko Weichert, Sebastian Hoberück, Jörg Kotzerke, Johannes Notni

**Affiliations:** 1 Institut für Pathologie Und Pathologische Anatomie, Technische Universität München, Trogerstraße 18, 81675 Munich, Germany; 2 Klinik Und Poliklinik für Nuklearmedizin, Universitätsklinikum Carl Gustav Carus an der Technischen Universität Dresden, Dresden, Germany

**Keywords:** Integrins, Beta8, Transforming growth factor beta, Positron emission tomography, Gallium-68, Preclinical imaging

## Abstract

**Purpose:**

As a major activator of transforming growth factor β (TGF-β), the RGD receptor αvβ8-integrin is involved in pathogenic processes related to TGF-β dysregulation, such as tumor growth, invasion, and radiochemoresistance, metastasis and tumor cell stemness, as well as epithelial-mesenchymal transition. The novel positron emission tomography (PET) radiopharmaceutical Ga-68-Triveoctin for in vivo mapping of αvβ8-integrin expression might enhance the prognosis of certain tumor entities, as well as support and augment TGF-β-targeted therapeutic approaches.

**Methods:**

Monomeric and trimeric conjugates of cyclo(GLRGDLp(*N*Me)K(pent-4-ynoic amide)) were synthesized by click chemistry (CuAAC), labeled with Ga-68, and evaluated in MeWo (human melanoma) xenografted SCID mice by means of PET and ex-vivo biodistribution. αvβ8-integrin expression in murine tissues was determined by β8-IHC. A human subject received a single injection of 173 MBq of Ga-68-Triveoctin and underwent 3 subsequent PET/CT scans at 25, 45, and 90 min p.i..

**Results:**

The trimer Ga-68-Triveoctin exhibits a 6.7-fold higher αvβ8-integrin affinity than the monomer (IC_50_ of 5.7 vs. 38 nM, respectively). Accordingly, biodistribution showed a higher tumor uptake (1.9 vs. 1.0%IA/g, respectively) but a similar baseline upon blockade (0.25%IA/g for both). IHC showed an intermediate β8-expression in the tumor while most organs and tissues were found β8-negative. Low non-target tissue uptakes (< 0.4%IA/g) confirmed a low degree of unspecific binding. Due to its hydrophilicity (log *D* = − 3.1), Ga-68-Triveoctin is excreted renally and shows favorable tumor/tissue ratios in mice (t/blood: 6.7; t/liver: 6.8; t/muscle: 29). A high kidney uptake in mice (kidney-to-blood and -to-muscle ratios of 126 and 505, respectively) is not reflected by human PET (corresponding values are 15 and 30, respectively), which furthermore showed notable uptakes in coeliac and choroid plexus (SUVmean 6.1 and 9.7, respectively, 90 min p.i.).

**Conclusion:**

Ga-68-Triveoctin enables sensitive in-vivo imaging αvβ8-integrin expression in murine tumor xenografts. PET in a human subject confirmed a favorable biodistribution, underscoring the potential of Ga-68-Triveoctin for mapping of αvβ8-integrin expression in a clinical setting.

## Background

β8-Integrin was first described in 1991 [1] as one of five β-integrins (β1, -3, -5, -6, and -8) pairing with the αv monomer, which is the only α integrin it dimerizes with. Although αvβ8 is comparable to other αv integrins (particularly αvβ3) in that it recognizes the extracellular matrix (ECM) protein vitronectin (Vn) by the arginine-glycine-aspartate (RGD) sequence contained therein, its uniqueness is manifested by the fact that unlike αvβ3, αvβ5, or αvβ1, transmembrane αvβ8 does not exert adhesive forces, i.e., does not promote cell binding to Vn [2]. Hence, unlike other integrins which transmit physical forces and thereby enable the adhesion of cells to ECM proteins, αvβ8 appears to be involved mainly into signaling.

Mounting experimental evidence suggested that explaining the biological role and significance of αvβ8-integrin requires, in essence, a closer view on a family of mammalian cytokines called transforming growth factor beta (TGF-β1 and -3, herein referred to as TGF-β) [3]. TGF-β is produced by almost any cell type and secreted into the extracellular space, albeit not as a free protein ligand capable of binding to its respective receptor, but as a "protected", i.e., inactive, aggregate with another inhibitory protein referred to as latency-associated peptide (LAP). This aggregate, called small latency complex (SLC), is often covalently linked to the ECM by another protein called latent TFG-β binding protein (LTBP), forming the so-called large latent complex (LLC). An essential functionality of αv-integrins (above all, αvβ8, and αvβ6) is their ability to release TGF-β from its complex with LAP by binding to a RGD sequence contained therein [4, 5]. Again, αvβ8-integrin displays a unique mode of action. While a pulling force exerted by αvβ6-integrin distorts the structure of LAP and exposes TGF-β to its receptor [6, 7], αvβ8 achieves a similar result by dragging the SLC into close proximity of matrix metalloprotease 14 (MMP14, synonym: MT1-MMP), which cleaves LAP and thereby generates free TGF-β [8]. Taken together, expression of αvβ8-integrin first and foremost enables cells to liberate TGF-β from its latent complexes in the extracellular space, and αvβ8 expression is therefore closely connected to TGF-β-related signaling and its role in pathogenesis, particularly of fibrosis and cancer [9].

Generally, TGF-β acts as a growth suppressor onto normal cells and may function as a tumor suppressor [10]. For instance, compared to normal airway epithelium, a low αvβ8-integrin expression was found in epithelial lung cancers, which apparently supports its progression because low αvβ8 results in reduced TGF-β levels and consequently derogated tissue homeostasis [11]. However, tumor cells may also escape the growth-inhibiting effect of TGF-β by means of altered downstream pathways, for instance, a Ser-15 mutation on p53 [12] or a loss of Smad4 [13]. Hence, any means of increasing the concentration of activated TGF-β in their surrounding, for instance by αvβ8-integrin upregulation, is assumed to give such cells a growth advantage over normal cells, turning TGF-β into a tumor growth promoter [9]. For TGF-β-resistant tumors, a concomitant αvβ8 upregulation must therefore be expected to promote invasion and metastasis, particularly because TGF-β also stimulates angiogenesis, epithelial-mesenchymal transition (EMT), cell motility, tumor cell stemness, and colonization of the metastatic niche [14]. Such pathogenic mechanisms have, for example, been detailed for glioblastoma (GBM) [15] and prostate cancer [16] cell lines, and for astrocytes which may control the angiogenic activity of adjacent endothelial cells by αvβ8-integrin expression-mediated regulation of local TGF-β levels [17]. In addition, other αvβ8-dependent signalling axes, e.g., involving RhoGDI1, may also be relevant for pathogenesis [18].

In view of its multifaceted role in human pathology and oncogenesis, we anticipate a substantial scientific and clinical value for in-vivo mapping of physiological and pathological αvβ8-integrin expression patterns. For this purpose, we developed ^68^Ga-Triveoctin, a ^68^Ga-labeled trimer of the αvβ8 selective octapeptide c(GLRGDLp(*N*Me)K) [19] suitable for imaging of αvβ8-integrin expression by means of positron emission tomography (PET).

## Methods

### General

Synthesis and characterization of Triveoctin is described in the Additional file 1. The integrin affinities were determined by a solid-phase binding assay, applying a previously described protocol [20]. β8 immunohistochemistry stainings were done as described [18]. All animal studies have been performed in accordance with general animal welfare regulations in Germany and the institutional guidelines for the care and use of animals. ^68^Ga radiolabeling [21], cultivation of MeWo cells and generation of respective subcutaneous xenografts [18], determination of *n*-octanol/phosphate-buffered saline (PBS) distribution coefficients (log *D*) and ex-vivo biodistribution studies [22], and µPET imaging [23] were done as described previously in detail (a brief summary is provided in the following).

### Radiochemistry and preclinical studies

For fully automated ^68^Ga labeling, non-processed eluate of a ^68^Ge/^68^Ga-generator with a SnO_2_ matrix (by IThemba LABS, SA; 1.25 mL, 1 M HCl, eluted ^68^Ga activity approx. 700 MBq) was adjusted to pH 2 by adding HEPES buffer (400 µL of a 2.7 M solution), used to label 2 nmol of Triveoctin or TRAP-AvB8 for 3 min at 95 °C, which was purified by solid-phase extraction using a SepPak® C8 light cartridge (*Waters*). Radiochemical yields were 95.8 ± 1.3% (*n* = 10), referring to the final product after purification. Quality control was performed by radio-TLC with citrate buffer, confirming > 99% purity (referring to absence of non-complexed ^68^Ga, see Figure S3). Distribution coefficients (log *D*) were determined by shake-flask method using n-octanol and PBS.

MeWo cells (ATCC®, HTB-65™) were grown at 37 °C under 5% CO_2_ atmosphere in DMEM/HAM (*Biochrom*, Berlin, Germany) with 10% fetal calf serum (*Thermo Fisher*). Approx. 10^7^ cells were subcutaneously injected with Matrigel® (*Corning*, #354262) into the right shoulder of 6–8-week-old female CB17 severe combined immunodeficiency (SCID) mice, which were used about 2–3 weeks later for PET and biodistribution.

For ocular autoradiography and histology, a mouse was sacrificed 50 min after injection of 12 MBq ^68^Ga-Triveoctin. The eyes were excised, rinsed with PBS and embedded in *Sakura* Tissue-Tek®. After equilibrium had been reached at − 20 °C, lateral 50-μm cross sections were cut on a cryostat microtome (*Leica* CM1950) and thaw-mounted on SuperFrost Plus microscope slides. The slides were air-dried on a heating plate and exposed to an imaging plate from 45 min after sacrifice onwards overnight. The imaging plate was read out by a CR-35 Bio Scanner (*Raytest*). Gaussian smoothing was applied to the final images. The same sections were subsequently HE-stained in a standard automated process.

### PET imaging in human

Using a fully automated synthesis module, the eluate of a ^68^Ge/^68^Ga generator (by *Eckert & Ziegler*, Berlin, Germany; 0.1 M HCl, approx. 500 MBq ^68^Ga) was directly eluted into the reaction vial containing Triveoctin (35 µg) and adjusted to pH 4.5 with sodium acetate. After heating for 4 min at 95 °C, the mixture was passed over a solid-phase extraction cartridge (*Waters* Sep-Pak®light tC-18), which was purged with water (10 mL). Thereafter, ^68^Ga-Triveoctin was eluted with ethanol/water mixture (1:1 by volume, 1 mL), followed by isotonic saline (9 mL). The formulation containing approx. 5% ethanol was passed through a 0.22-µm filter into a sterile injection vial and dispensed for injection. Quality control was done by radio-HPLC using a *Shimadzu* system equipped with a column Chromolith® Performance RP18e (*Phenomenex*, Aschaffenburg, Germany), gradient 0–100% acetonitrile in water within 15 min, flow rate 1.4 mL/min, *t*_R_ = 8.53 min, and met the in-house specifications for ^68^Ga-labeled compounds (> 95%).

Application of ^68^Ga-Triveoctin was done according to §13/2b of the German Drug Act (Arzneimittelgesetz). A human subject received a single intravenous injection of ^68^Ga-Triveoctin (173 MBq, approx. 25 µg; for radiolabeling and QC, see Additional file 1). There were no adverse or clinically detectable pharmacologic effects. No significant changes in vital signs or the results of laboratory studies or electrocardiograms were observed. 25 min p.i., the patient underwent a list-mode PET/CT imaging protocol on a Biograph Vision 600 (*Siemens Healthineers*, Knoxville, USA). A standard low-dose CT was acquired from the whole body (X-ray tube current 10 mA, tube voltage 100 kV, spiral pitch factor 1.5, 3.0 mm slice thickness) and used for absolute scatter correction of the subsequent PET scan. The emission PET scan was acquired over 19 min using continuous bed motion with a speed of 2.2 mm s^–1^ for the legs and 1.4 mm s^–1^ for the remaining body. The PET scan was repeated 45 min p.i. without another CT scan. Another PET/CT imaging sequence was acquired 90 min p.i. as the subject had to leave the scanner for voiding. All scans were obtained during normal breathing. PET images were reconstructed using the TRueX algorithm with 4 iterations, 5 subsets, time-of-flight (TOF) application and without filtering. Resulting PET images had an image matrix size of 440 × 440 with a voxel size of 1.65 × 1.65 × 3.0 mm. The dosimetry values were calculated using OLINDA V1.1 [24] on the basis of the human PET data.

## Results

### Synthesis and affinity data

**Fig. 1:**
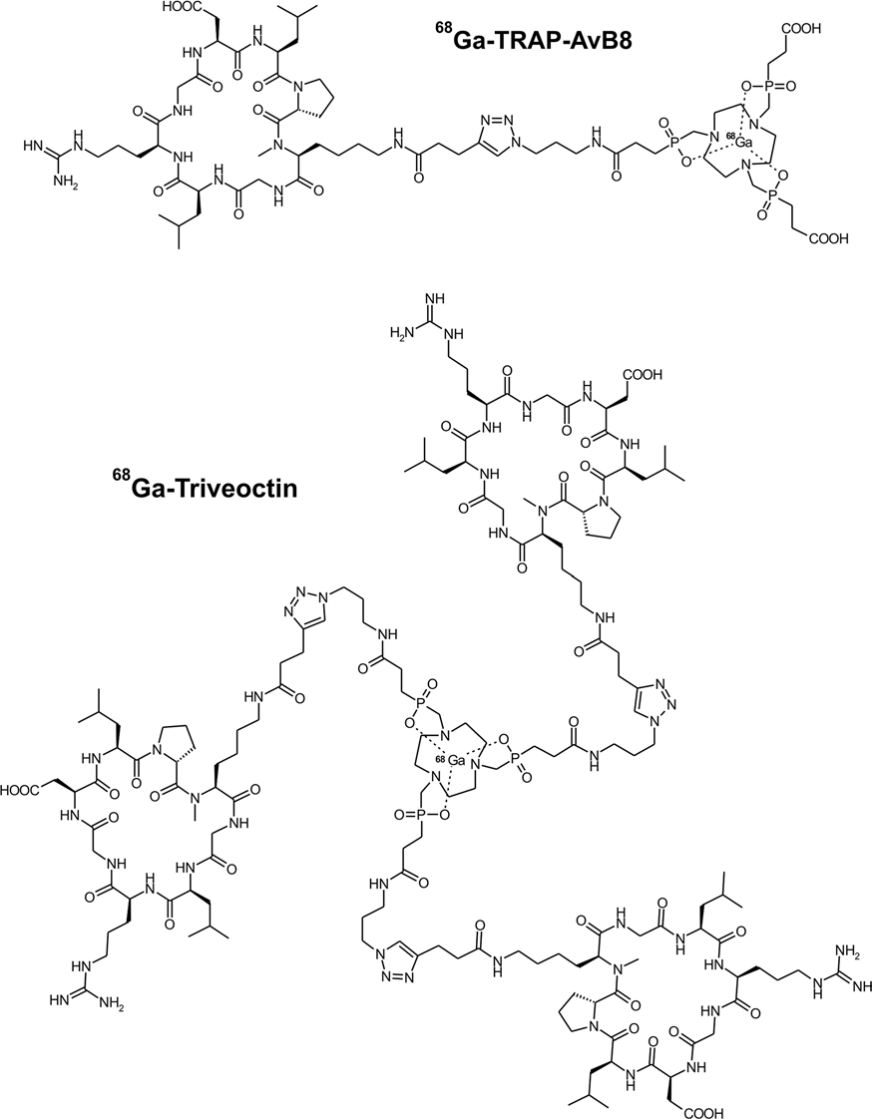
^68^Ga-TRAP-AvB8 and ^68^Ga-Triveoctin, two gallium-68-labeled conjugates of the peptide c(GLRGDLp(*N*Me)K) (AvB8) for in-vivo mapping of αvβ8-integrin expression by PET

Design of the ^68^Ga-peptides was done adopting a synthetically robust scheme [25] which has been successfully employed previously for elaboration of mono- and multimeric αvβ6-integin ligands [26] or PSMA inhibitors [27]. Azide-decorated derivatives of a TRAP chelator [28] (more precisely, 1,4,7-triazacyclononane-1,4,7-tris[methylene(2-carboxyethyl)]phosphinic acid) [29] were coupled to the respective alkyne-functionalized peptidic building block AvB8-pentynoic amide by means of click chemistry (CuAAC) [30]. Subsequent ^68^Ga labeling afforded the novel trimer ^68^Ga-Triveoctin (Fig. 1) and the previously described monomer ^68^Ga-TRAP-AvB8 [18] (for reaction schemes and details on syntheses, see Additional file 1).

In comparison with the linker-decorated peptide, the monomeric conjugate showed only approx. half of the biological activity (IC_50_ of 38 vs. 17 nM, respectively). Trimerization, however, resulted in a threefold and 6.7-fold higher target affinity for Ga-Triveoctin as compared to the neat peptide and the chelator monomer, respectively (Table 1).

**Table 1:** αvβ8-Integrin affinities (expressed as 50% inhibition concentrations, IC_50_) and *n*-octanol-PBS distribution coefficients (log *D*_7.4_). Affinities were determined using the non-radioactive ^69/71^Ga^III^ complexes, where applicable

Compound	IC_50_ (95% confidence interval) [nm]	log *D*_7.4_
^68^Ga-Triveoctin (trimer)	5.7 (3.6–9.0)	− 3.1 ± 0.1
^68^Ga-TRAP-AvB8 (monomer)	38 (25–58)	− 3.9 ± 0.1
AvB8-pentynoic amide	17 (12–24)	n/a

### Preclinical in-vivo characterization

**Fig. 2:**
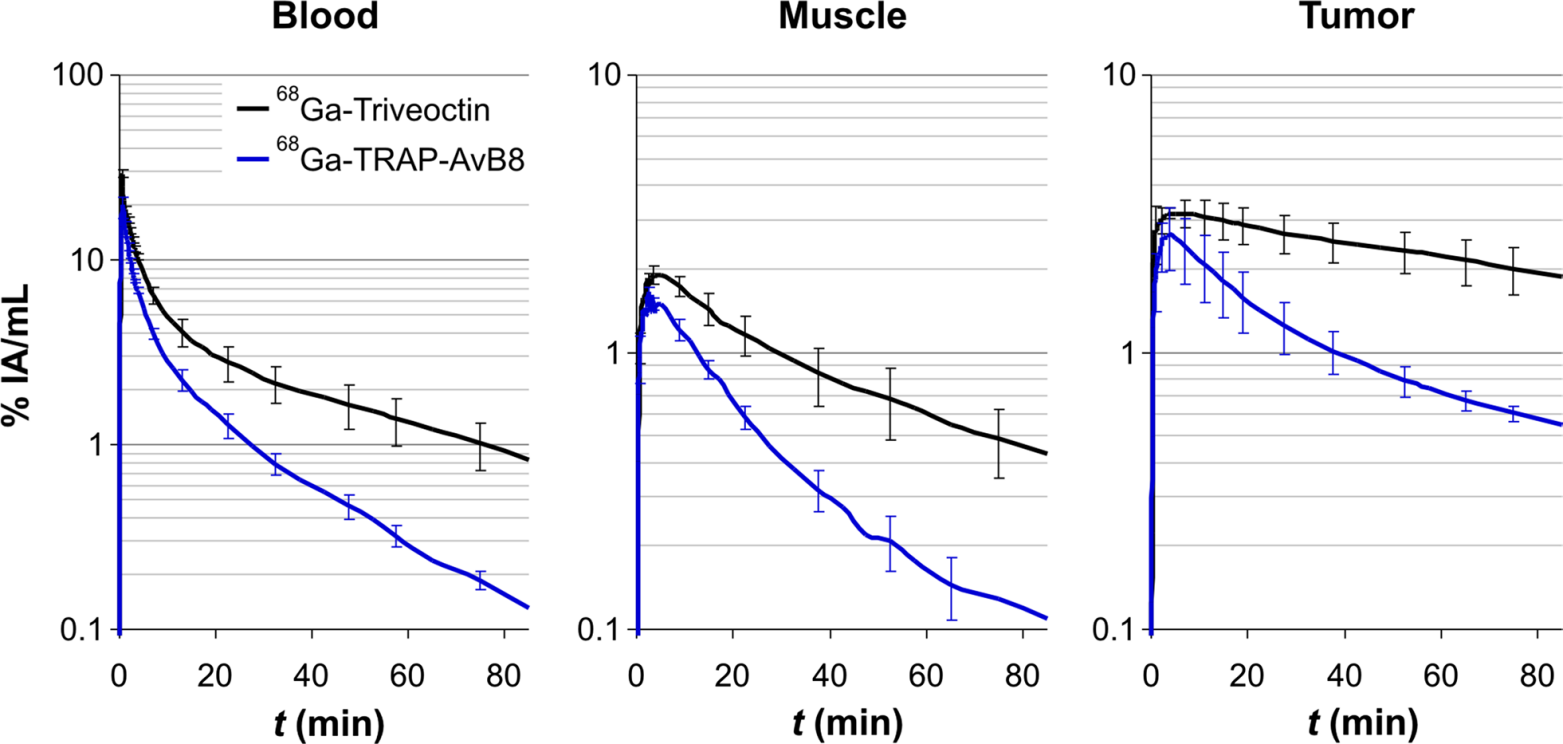
Kinetics for ^68^Ga-Triveoctin (black lines; 11 ± 1 MBq, 64 ± 13 pmol, 182 ± 30 MBq/nmol) and ^68^Ga-TRAP-AvB8 (blue; 11 ± 1 MBq, 151 ± 107 pmol, 93 ± 50 MBq/nmol), derived from dynamic PET data in MeWo-xenografted SCID mice (*n* = 3)

Consistent with its higher αvβ8-integrin affinity, dynamic PET data illustrate that the trimer ^68^Ga-Triveoctin shows a substantial better tumor retention than the monomer ^68^Ga-TRAP-AvB8 (Fig. 2). However, the trimer also exhibits a slower clearance from muscle and blood, which might be attributed to its larger molecular size and to its slightly lower hydrophilicity (Table 1).

**Fig. 3:**
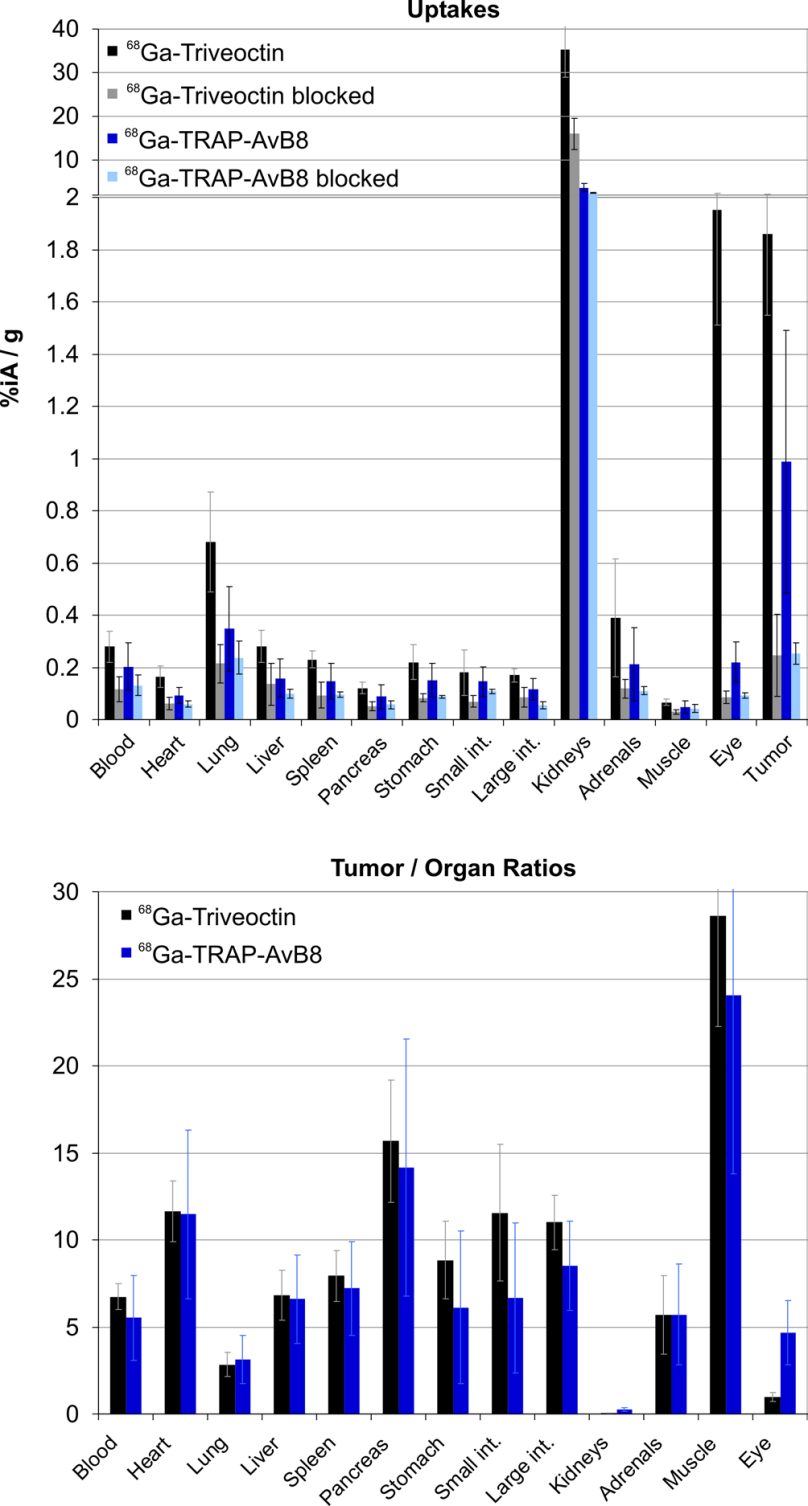
Biodistribution of ^68^Ga-Triveoctin, 60 min p.i. (43 ± 13 pmol, *n* = 6; blockade with 60 nmol Triveoctin injected 5 min prior to the radiopharmaceutical, *n* = 4) in MeWo-xenografted SCID mice, uptakes expressed as % injected dose per gram tissue; mean ± SD. Data for ^68^Ga-TRAP-AvB8 (control: 77 ± 24 pmol, *n* = 11; blockade: *n* = 3) were taken from the literature [18] and are shown for comparison. Data in numerical form are given in the Additional file 1: Table S1

This result is corroborated by biodistribution data (Fig. 3). Although the background uptake in all tissues is low for both tracers, a noticeably higher level is observed for ^68^Ga-Triveoctin. However, in terms of contrast, this is compensated for by an almost two times higher tumor uptake, ultimately resulting in somewhat larger tumor-to-organ ratios of the trimer (Fig. 3).

**Fig. 4:**
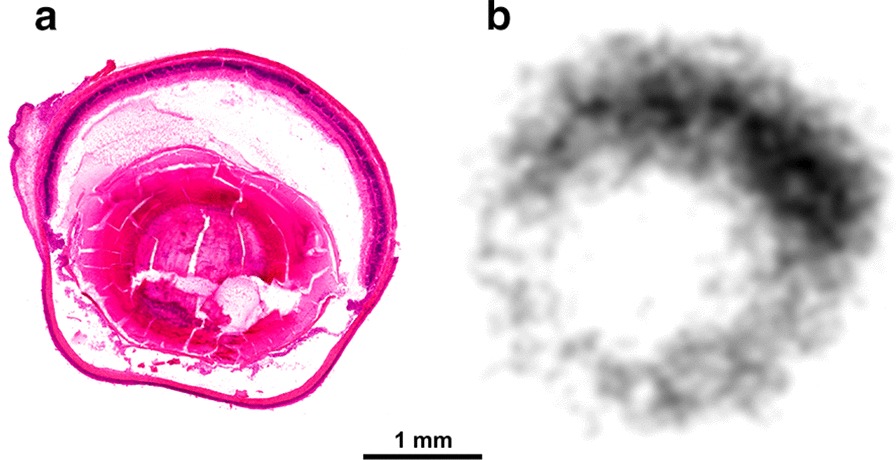
HE staining (**a**) and corresponding autoradiography image (**b**) of a representative sagittal mouse eye cryoslice (50 µm), 50 min after administration of ^68^Ga-Triveoctin

Interestingly, there are striking exceptions, namely the lung and the eye, showing blockable uptake of ^68^Ga-Triveoctin but not of ^68^Ga-TRAP-AvB8. Autoradiography of a lateral eyeball cryoslice indicated that the activity is apparently neither accumulated in the vitreus nor the lens or the cornea but concentrates in the area of the dorsal layers (Fig. 4). Since astrocyte-expressed αvβ8-integrin plays an essential role for the vascularization of the developing mouse retina [31], a persistent expression in the mature retina might be responsible for the observed uptake in the eyeballs.

**Fig. 5:**
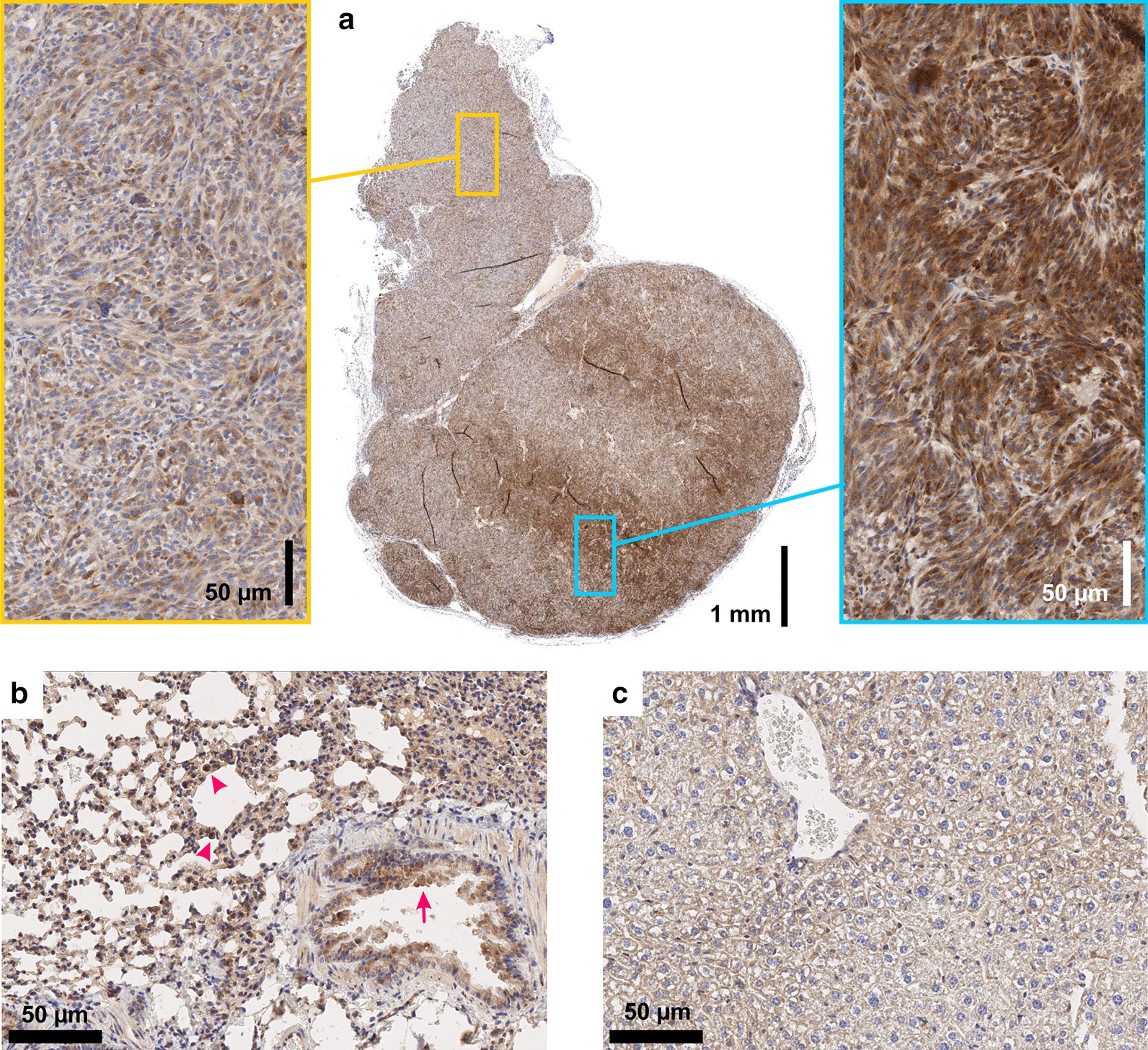
β8 integrin immunohistochemistry (IHC) of MeWo tumor (**a**), lung (**b**), and liver tissue (**c**) of the same animal used for comparative PET scans (Fig. 6). Magnifications in yellow and cyan frames show MeWo areas with low and high cellular β8 integrin expression, respectively. A slight β8 expression is observed in the bronchiolar epithelium of lung (arrows) and alveolar macrophages (arrowheads). No β8 expression in liver tissue. Note that β8 integrin dimerizes only with the ubiquitous αv monomer, which is why β8 is limiting and indicative for actual αvβ8 distribution

In addition, β8 immunohistochemistry (IHC) validation of target expression confirms that there is a non-negligible αvβ8 expression density on epithelial cells of the lung (Fig. 5b), which apparently can be detected in the PET by the trimer ^68^Ga-Triveoctin but not with the monomer because of its lower affinity.

**Fig. 6:**
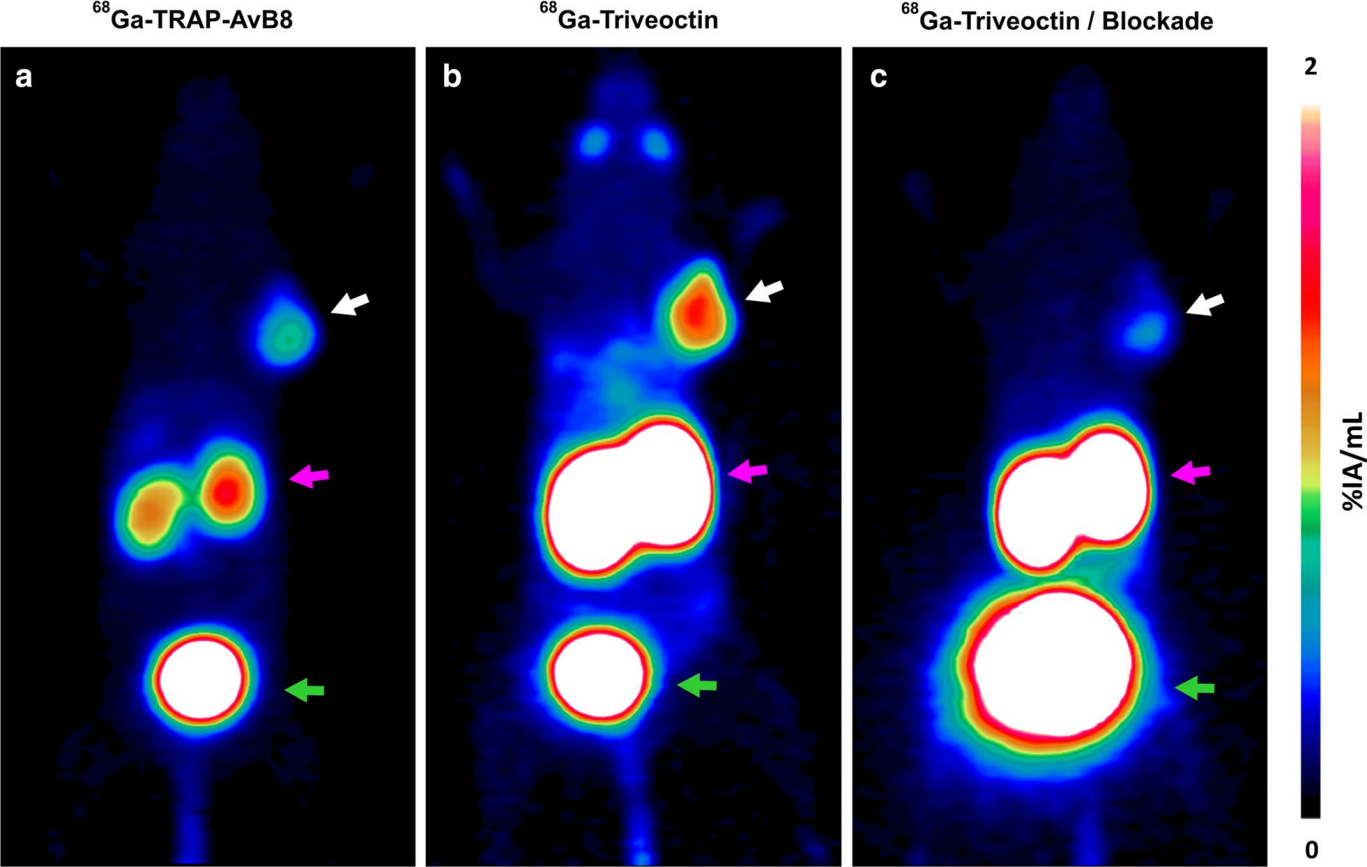
Representative PET images (maximum intensity projections, 60 min p.i., OSEM3D reconstruction) of a SCID mouse bearing a subcutaneous MeWo xenograft (human melanoma, positions indicated by white arrow), using ^68^Ga-TRAP-AvB8 (**a** 12 MBq, 35 pmol, 350 MBq/nmol) and ^68^Ga-Triveoctin (**b** 9 MBq, 25 pmol, 350 MBq/nmol). Blockade (**c**) was done by administration of 60 nmol Triveoctin, 10 min prior to the radiopharmaceutical. Purple and green arrows indicate presence of activity in kidneys and urinary bladder, respectively

Figure 5a furthermore shows that αvβ8-integrin density in a MeWo xenograft can be quite heterogeneous, while it is not entirely clear which factors are determining the observed local differences. Irrespective of that, the given example shows that the higher sensitivity of ^68^Ga-Triveoctin enables imaging αvβ8-integrin in tumor regions with low expression, which ^68^Ga-TRAP-AvB8 is not capable of. Figure 6 shows the PET images of the same animal used for IHC (Fig. 5), while the orientation of the slice analyzed by IHC corresponds to the PET position. Obviously, ^68^Ga-TRAP-AvB8 allows only for delineation of the central part of the tumor with high β8 expression (Fig. 6a), whereas ^68^Ga-Triveoctin PET readily reproduces the entire, pear-shaped tumor mass (Fig. 6b). Both tracers are, as expected, not significantly accumulated in β8-negative tissues, such as liver (Fig. 5c).

The µPET furthermore visualizes that compared to the kidney uptake of ^68^Ga-TRAP-AvB8 (averagely 3.6%IA/g, see Additional file 1: Table S1), a substantially higher value is observed for ^68^Ga-Triveoctin (35%IA/g), which is not reduced completely upon blockade but only diminished by slightly more than half (16%IA/g remaining). A substantial fraction of the remaining uptake could be caused by the larger molecular size, i.e., to less advanced renal clearance observed at the same time point (60 min p.i.) because of slower excretion kinetics. The main reason for the elevated kidney uptake might however be the expression of αvβ8-integrin on mural mesangial cells [32, 33]. The markedly higher uptake of ^68^Ga-Triveoctin can therefore be interpreted as another sign of its target sensitivity, i.e., its superior capability of visualizing mesangial αvβ8-integrin expression in mice.

### ^68^Ga-Triveoctin PET in human

**Fig. 7:**
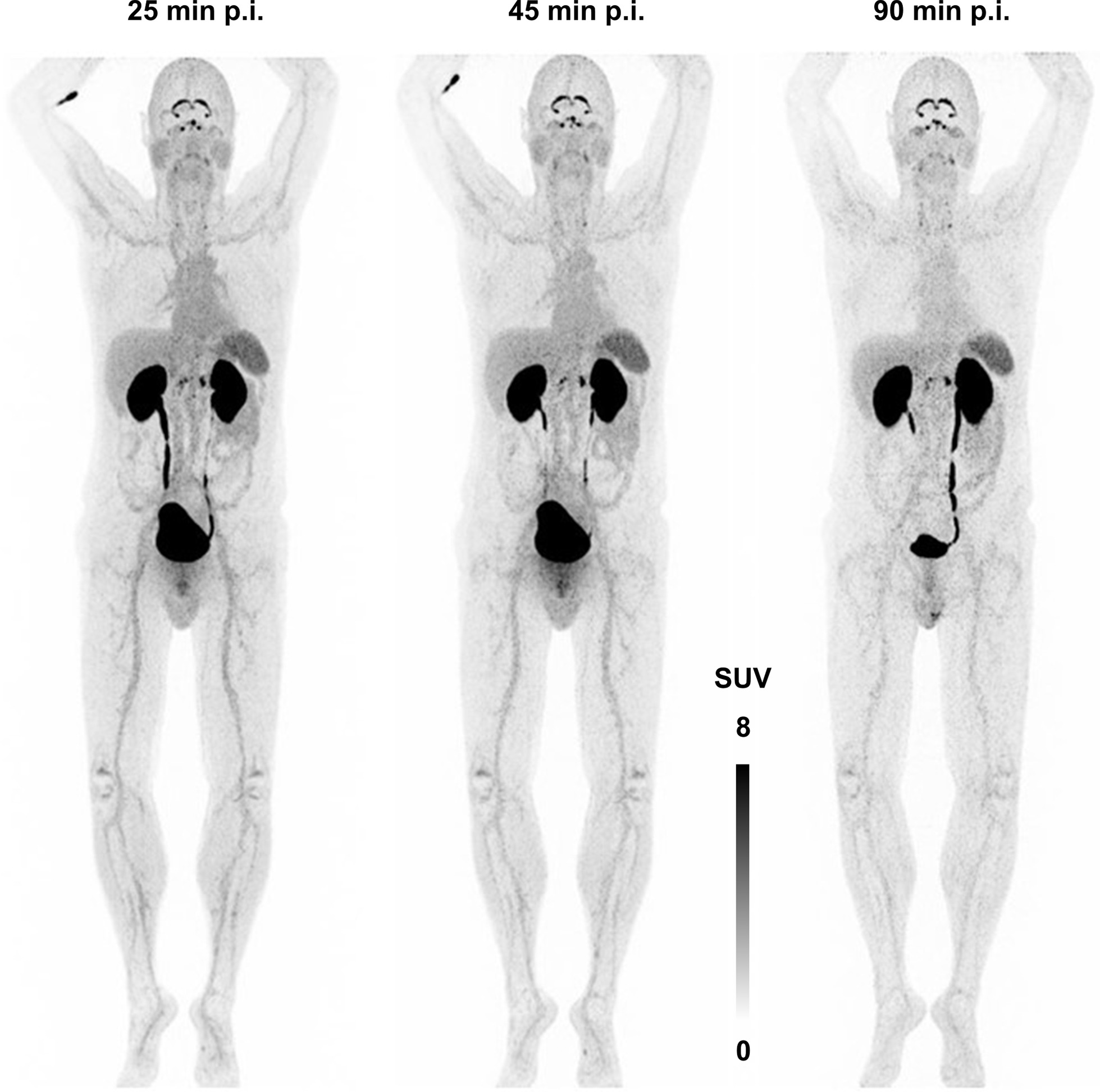
^68^Ga-Triveoctin PET (173 MBq, anterior maximum intensity projections) in human

While ^68^Ga-Triveoctin showed only a low soft tissue uptake in a human subject, notable tracer accumulations with descending intensity were observed in kidneys, urinary bladder, choroid plexus, infraadrenal (likely a ganglia), spleen, gastric mucosa, retina, liver, larger vessels, salivary glands and intestine (Fig. 7). As expected, renal uptake decreased over time. The tracer accumulation in the choroid plexus, the coelic ganglia, the spleen, the gastric mucosa, the retina, and the liver showed no relevant change during the imaging period, while there was a slight increase in the intestinal uptake and a decrease in the retained vascular activity (Table 2).

**Table 2:** Standard uptake values (SUV_mean_; SUV_max_ in parentheses) for selected areas of a PET scan in human (Fig. 7)

Organ	25 min p.i		45 min p.i		90 min p.i	
Blood (thoracic aorta)	2.43	(3.49)	1.85	(3.22)	1.61	(3.02)
Heart	2.27	(4.11)	1.88	(3.43)	1.48	(2.81)
Lung (avg.)	0.72	(1.09)	0.64	(1.24)	0.50	(1.03)
Liver	2.25	(4.26)	1.75	(3.19)	1.94	(3.74)
Spleen	3.97	(5.78)	3.97	(5.79)	3.81	(5.64)
Pancreas	1.75	(3.19)	1.64	(3.21)	1.30	(2.35)
Stomach	2.84	(5.01)	2.62	(4.99)	2.22	(4.08)
Small intestine	2.20	(4.06)	2.05	(3.91)	2.07	(4.18)
Large intestine (ascending)	1.50	(3.07)	1.24	(2.48)	1.16	(2.11)
Kidneys (avg.)	43.7	(78.2)	23.4	(52.8)	24.5	(54.9)
Urinary bladder	93.2	(129)	126	(206)	116	(172)
Adrenals (avg.)	2.27	(4.12)	2.15	(3.70)	2.10	(3.76)
Muscle	0.78	(1.45)	0.71	(1.40)	0.82	(1.60)
Eye (avg.)	2.11	(3.82)	2.34	(4.35)	2.64	(4.78)
Coeliac plexus (avg.)	6.51	(12.3)	6.80	(12.2)	6.07	(11.3)
Choroid plexus	13.7	(26.9)	11.1	(20.8)	9.71	(17.4)

Based on the data shown in Table 2 which implicates renal excretion with a biological half life of 1.8 h, and an assumed urinary bladder residence of 0.3 h, dosimetry calculations using OLINDA yielded a moderate effective dose (ICRP 60) of 3.00E−02 mSv/MBq. Assuming that an activity of 100 MBq ^68^Ga-Triveoctin is sufficient for a PET scan at 30–60 min p.i., up to 6 investigations could be performed without exceeding the 20 mSv limit.

## Discussion

### Radiotracer design: why a trimer?

Several studies have shown quite consistently that combining more than a single copy of a given targeting molecule (frequently referred to as multimerization) reliably increases the overall avidity of such constructs (for examples featuring RGD peptides, see refs [26, 34–39]), and ligand multiplicity is normally correlated to target uptake [40] (we are only aware of a single counterexample reported) [38]. However, multimerization does not necessarily improve the image quality or the overall diagnostic value, most likely because the larger molecular size and a different polarity profile (as compared to the respective monomers) frequently spoils the fundamental pharmacokinetic properties, for instance, increases unspecific organ uptake and -retention, or alters the excretion route and -velocity [40]. It is furthermore obvious that the hydrophilicity-enhancing effect of certain highly polar radiometal chelates, which can improve the overall pharmacokinetics of their conjugates with non-polar peptides [41], is less pronounced for multimers comprising a single chelate and several peptide moieties. Multimers of comparably nonpolar peptides thus may show unsuitable pharmacokinetics because of overall insufficient hydrophilicity [26]. The polarity of c(GLRGDLp(*N*Me)K) itself must therefore be regarded as the main reason why ^68^Ga-Triveoctin possesses a suitable hydrophilicity (log *D*_OW_ = − 3.1), and its higher αvβ8-integrin affinity and subtype selectivity can actually translate into favorable imaging characteristics. The ^68^Ga chelator TRAP [21] was chosen as a scaffold not only because its structure allows for facile elaboration of trimers [25, 30], but also because it tolerates comparably high concentrations of frequently occurring metal ion contaminants in generator eluates and ^68^Ga labeling solutions, such as Fe^III^ [42], Zn^II^, and Cu^II^ [43], and therefore enables highly efficient and robust radiolabeling procedures [44].

### Preclinical versus human αvβ8-integrin PET

Comparison of human PET data with murine ex-vivo distribution reveals several discrepancies, most strikingly, regarding eye uptake, which is high in mice but insignificant in human. Whether this is related to species or subject age (in relation to their typical life spans) cannot be answered on the basis of the existing data, but eye uptake seems to be an artifact of the preclinical experiments. Likewise, the kidney-to-blood and -to-muscle ratios at 90 min p.i. in human (approx. 15 and 30, respectively) are about 8 and 16 times, respectively, lower than in mice (126 and 505, respectively). The relatively high kidney uptake in mice thus also appears to be a mouse artifact, which might in part be related to the expression of αvβ8-integrin on mural mesangial cells [32, 33], but also to different excretion kinetics of the fairly large molecule. On the other hand, a known β8-integrin expression in normal human lung epithelial cells [11] is reflected by the mouse model (Fig. 4b). Although this leads to elevated tracer uptake per tissue weight in the mouse lung, no corresponding per-volume signal is observed in human PET because of the high spatial dilution of the nuclide owing to the high porosity of intact lung tissue.

### Implications and clinical perspective of αvβ8-integrin imaging

The existing insights into the expression and functions of αvβ8-integrin, which were gained mainly from cell studies and histological data, do not allow for a valid conclusion whether or not this receptor might be suited for a "if you can see it, you can treat it" radionuclide imaging and -therapy approach similar to the highly popular and successful sst2- and PSMA-targeted theranostics [45]. While there is yet no systematic, large-cohort-based data on αvβ8 expression for a comprehensive range of tumor types or conditions, a recent histological study indicated a high proportion of β8-positive tumor cells in various carcinomas (ovarian, uterine endometrioid, skin, in situ breast ductal, gastric adenocarcinoma, and particularly oral squamous cell carcinoma), although based on only relatively small numbers of patient specimen (3–22 per entity) [46]. Theranostic applications targeting αvβ8-integrin therefore do not seem to be unrealistic but remain to be thoroughly investigated.

Beyond the classic theranostic paradigm, the involvement of αvβ8 in major oncogenic pathways, such as TGF-β1 signaling and EMT, might ultimately render αvβ8-PET useful for clinical reasoning and personalized medicine, such as for cancer prognosis and stratification of patients for certain chemotherapies. In view of a high relevance of PET imaging of the TGF-β signaling pathway adressing downstream targets [47], αvβ8-PET might augment and/or substitute such approaches. According to the current state of knowledge, it appears furthermore plausible that tumors with an elevated αvβ8 level could be resistant to TGF-β-mediated growth suppression (otherwise, they would inhibit their own progression and thus could not have developed in the first place). Such tumors, for which TGF-β acts as a growth promoter [10], should be susceptible to treatment with TGF-β inhibitors. αvβ8-Integrin PET might therefore be useful for selecting appropriate patients for anti-TGF-β therapies [14, 48]. Nishimura and colleagues recently went even further and proposed that rather than targeting latent or free TGF-β itself, inhibition of ligands and receptors involved in TGF-β activation, and particularly of αvβ8-integrin, might allow for a TGF-β-directed therapy with an improved cell type and context specificity [46]. This approach could potentially help to mitigate the systemic toxicity arising from a global loss of the essential homeostatic functions of TGF-β upon blockade [49, 50]. Likewise, αvβ8-integrin itself has been suggested as a drug target, e.g., for treatment of GBM [15] as well as to overcome a gefitinib resistance of hepatic cancer [51] or radiochemoresistance of pancreatic ductal adenocarcinoma [52]. In case of a future application of anti-αvβ8-agents for such purposes, patient selection based on αvβ8-specific noninvasive imaging obviously makes sense. In this respect, the comparably low overall dose allowing for repeated ^68^Ga-Triveoctin PET (up to 6 scans below the 20 mSv limit) might even allow for in-depth studies of target expression as a response to therapy.

## Conclusion

The αvβ8-integrin PET diagnostic ^68^Ga-Triveoctin was obtained by trimerization of the cyclic peptide cyclo(GLRGDLp(*N*Me)K) on the TRAP chelator scaffold. Because of its high αvβ8-integrin affinity, the tracer enabled sensitive in-vivo imaging in murine tumor xenografts. PET in human confirmed a favorable biodistribution with low background, underscoring the potential of ^68^Ga-Triveoctin for future clinical investigation of αvβ8-integrin expression, e.g., for conditions associated with TGF-β dysregulation.

**Table 3:** Dose estimates for ^68^Ga-Triveoctin, calculated with OLINDA V1.1 based on the organ residence times shown in Table 2

Organ	Dose (mGy/MBq)
Adrenals	2.69E−02
Brain	5.23E−03
Breasts	5.46E−03
Gallbladder wall	1.00E−02
LLI wall	2.08E−02
Small intestine	3.52E−02
Stomach wall	1.95E−02
ULI wall	1.57E−02
Heart wall	1.52E−02
Kidneys	2.60E−01
Liver	2.43E−02
Lungs	7.83E−03
Muscle	7.23E−03
Ovaries	1.13E−02
Pancreas	2.01E−02
Red marrow	6.48E−03
Osteogenic cells	8.89E−03
Skin	5.60E−03
Spleen	4.42E−02
Testes	8.14E−03
Thymus	6.17E−03
Thyroid	5.72E−03
Urinary bladder wall	3.42E−01
Uterus	1.57E−02
Total body	9.41E−03

## Supplementary information

**Additional file 1:** Synthesis and analytical data for Triveoctin, radio-TLC for ^68^Ga-Triveoctin, and small-animal biodistribution data for ^68^Ga-Triveoctin in numerical form (table).

## Data Availability

The datasets used and/or analyzed during the current study are available from the corresponding author on reasonable request.
