# Supplementary material for: Tracking a TGF-β activator in vivo: sensitive PET imaging of αvβ8-integrin with the Ga-68-labeled cyclic RGD octapeptide trimer Ga-68-Triveoctin
Source: EJNMMI Res. 2020 Oct 31;10:133. doi: 10.1186/s13550-020-00706-1 (PMC7603442; doi:10.1186/s13550-020-00706-1)
Supplement: Supplementary file 1 — Additional file 1: Synthesis and analytical data for Triveoctin, radio-TLC for 68Ga-Triveoctin, and small-animal biodistribution data for 68Ga-Triveoctin in numerical form (table). [file 13550_2020_706_MOESM1_ESM.pdf]

Supporting Information

Tracking a TGF-β Activator In Vivo: Sensitive PET Imaging of αvβ8-Integrin with the Ga-68 Labeled Cyclic RGD Octapeptide Trimer Ga-68-Triveoctin

Neil Gerard Quigley, Katja Steiger, Frauke Richter, Wilko Weichert, Sebastian Hoberück, Jörg Kotzerke, Johannes Notni

Synthesis

General

Unless otherwise noted, all commercially available reagents and solvents were of analytical grade and were used without further purification. Protected amino acids were purchased from *IRIS Biotech* (Germany). $Cu(OAc)_2 \cdot H_2O$, 4-pentynoic acid, diisopropylamine (DIPEA) and sodium ascorbate were purchased from *Sigma Aldrich* (Darmstadt, Germany). 1,4,7-triazacyclononane-1,4,7-triacetic acid (NOTA) was purchased from *Chematech* (Dijon, France). HATU was obtained from *Bachem Holding AG* (Bubendorf, Switzerland). HOBt hydrate was obtained from *Carbolution* (St. Ingbert, Germany). TRAP(azide)$_3$ was synthesized as described previously [1]. Semi-preparative reversed-phase HPLC was performed using a *Waters* system: *Waters 2545* (Binary Gradient Module), *Waters SFO* (System Fluidics Organizer), *Waters 2996* (Photodiode Array Detector) and *Waters 2767* (Sample Manager). Separations were performed using a *YMC* Pack ODS-A, 5 µm, 250×20 mm column with a flow rate of 16 mL/min of water (0.1 % v/v trifluoroacetic acid) and acetonitrile (0.1 % v/v trifluoroacetic acid). Analytical HESI-HPLC-MS (heated electrospray ionization mass spectrometry) was performed on a LCQ Fleet (*Thermo Scientific*) with a connected UltiMate 3000 UHPLC focused (*Dionex*) on C18-columns: S1: Hypersil Gold aQ 175 Å, 3 µm, 150×2.1 mm (for 8 or 20 min measurements); S2: Accucore C18, 80 Å, 2.6 µm, 50×2.1 mm (for 5 min measurements) (*Thermo Scientific*). Linear gradients (5%–95% acetonitrile content) with water (0.1% v/v formic acid) and acetonitrile (0.1% v/v formic acid) were used as eluents. AvB8-pentynoic amide and TRAP-AvB8 were synthesized as described in the literature [2].

Triveoctin

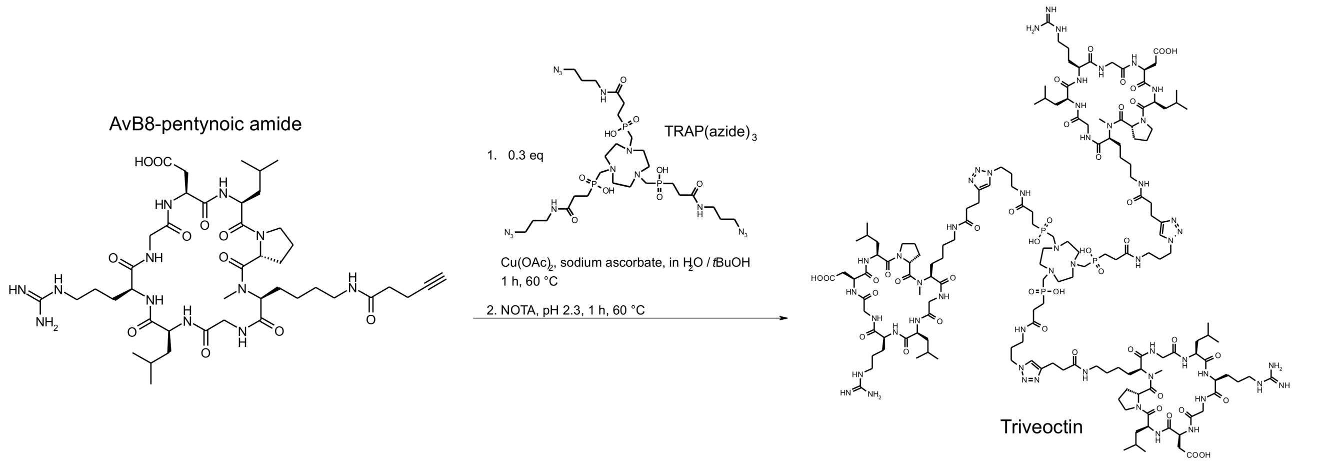

CuAAC coupling and Cu removal steps were monitored by mass spectroscopy analysis. AvB8-pentynoic amide (45.6 mg, 49 µmol, 3.3 eq), sodium ascorbate (147 mg, 742 µmol, 50 eq) and TRAP(azide)$_3$ (12.3 mg, 14.8 µmol, 1 eq) were dissolved in a solution of *tert*-butanol: Water, 1:3 (1 mL). Copper(II) acetate (3.6 mg, 17.9 µmol, 1.2 eq) was added to the solution and immediately a brown precipitate formed, which dissolved upon vortexing (1 min), resulting in a transparent green solution. The solution was heated in a water bath for 1 h at 60 °C without stirring. Cu removal from the chelator cavity and sequestration of any unbound Cu was carried out as described [1] by addition of NOTA (134 mg, 462 µmol, 30 eq) and adjustment of the solution to pH 2.2–2.4 with 1 M aq HCl. The solution was heated in a water bath for 1 h at 60 °C without stirring. Then, the reaction solution was directly subjected to preparative RP-HPLC purification (linear gradient: 25–60 % MeCN in H_2O containing 0.1 % trifluoroacetic acid in 15 min, t_R = 11.9 min). Evaporation and lyophilization of eluate-containing fractions yielded Triveoctin (15.1 mg, 4.2 µmol, 27 %) as a colorless solid, MW (calcd for $C_{156}H_{264}N_{51}O_{42}P_3$): 3619.06. MS (ESI, positive mode): *m/z* = 1809.8 $[M+2H^+]^{2+}$, 1206.8 $[M+3H^+]^{3+}$, 905.5 $[M+4H^+]^{4+}$, 724.5 $[M+5H^+]^{5+}$. ^{nat}Ga-Triveoctin for determination of integrin affinities was prepared by mixing equimolar amounts of aqueous solutions of Triveoctin and $Ga(NO_3)_3$ (2 mM, 100 µL each), followed by lyophilization. MW (calcd for $C_{156}H_{264}N_{51}O_{42}P_3Ga$): 3685.76. MS (ESI, positive mode, ^{69}Ga isotope peaks only): *m/z* = 1841.9 $[M+2H^+]^{2+}$, 1227.6 $[M+3H^+]^{3+}$, 921.2 $[M+4H^+]^{4+}$, 737.3 $[M+5H^+]^{5+}$ (for MS spectra, see Figure S1 and S2).

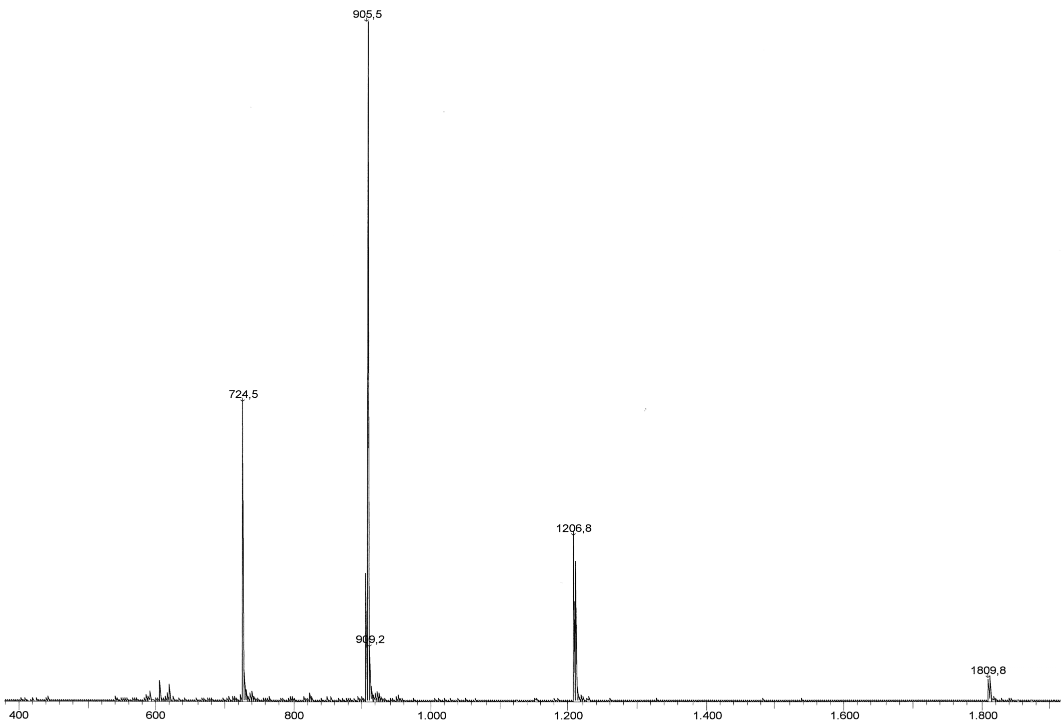

Figure S1: Mass spectrum (ESI, positive mode) for Triveoctin.

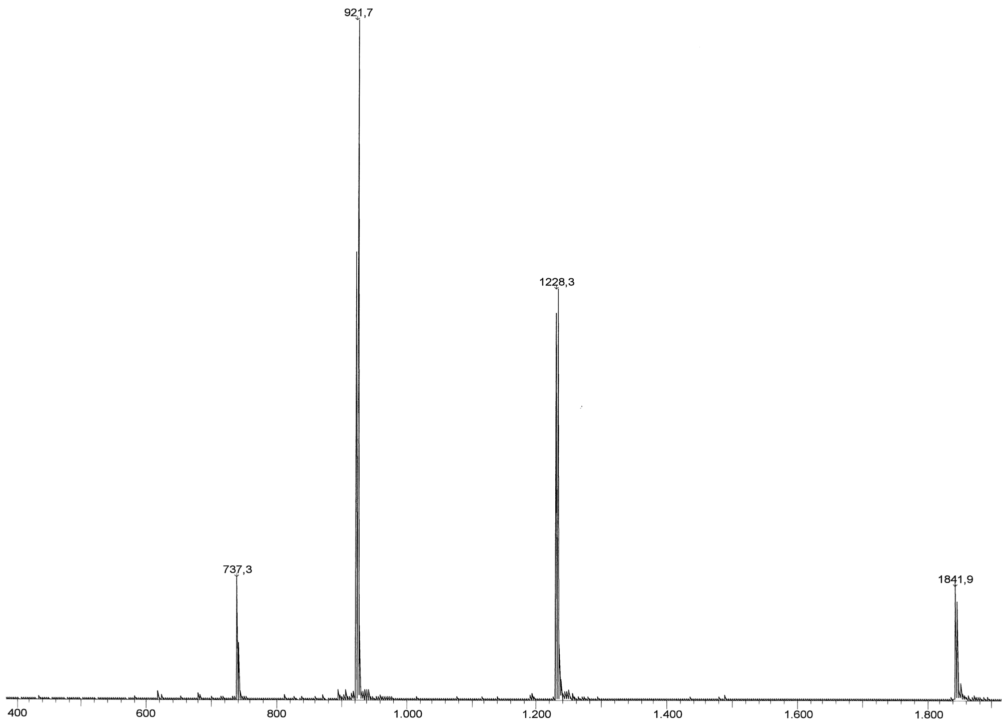

Figure S2: Mass spectrum (ESI, positive mode) for ^{nat}Ga-Triveoctin.

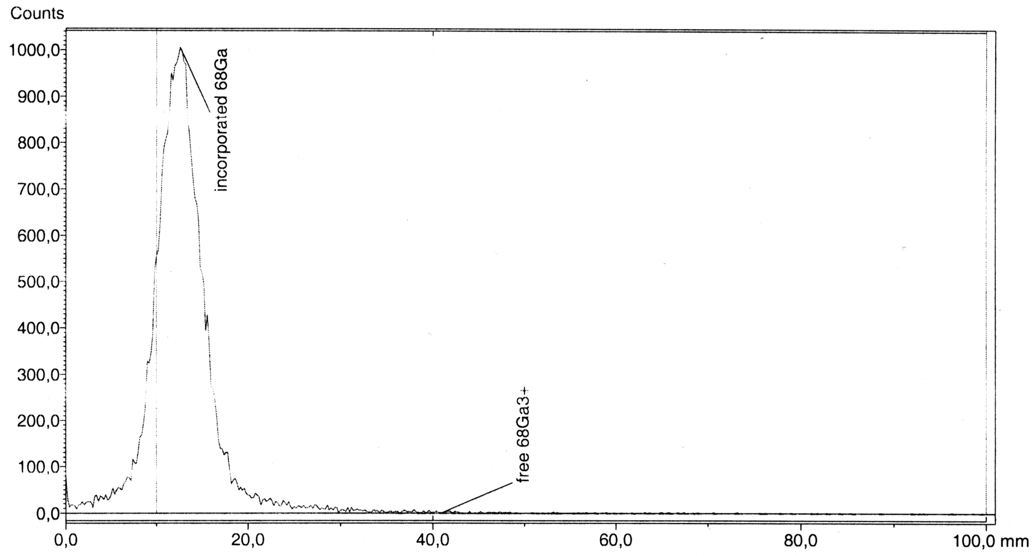

Regions:

Name	Start (mm)	End (mm)	Retention (RF)	Height (Counts)	Area (Counts)	%ROI (%)	%Total (%)
incorporated 68Ga	0,0	40,0	0,0	1006,0	30509,0	99,53	99,53
free 68Ga3+	40,0	100,0	0,3	5,0	143,0	0,47	0,47
2 Peaks					30652,0	100,00	100,00

Figure S3: Exemplary radio-TLC for ^{68}Ga-Triveoctin (stationary phase: Agilent ITLC® chromatography paper; mobile phase: 0.1 M aq. sodium citrate, adjusted to pH 5.5).

Table S1: Biodistribution data (60 min p.i.) for ^{68}Ga-Triveoctin without (n = 6) and with (n = 4) addition of 60 nmol of Triveoctin. Data are given as averages ± standard deviation. %IA/g = percent injected activity per gram tissue.

Organ/Tissue	^{68}Ga-Triveoctin		+ 60 nmol cold
	%IA/g	tumor/organ ratio	10 min prior to activity
Blood	0.28 ± 0.06	6.7 ± 0.7	0.12 ± 0.05
Heart (myocard)	0.16 ± 0.04	11.7 ± 1.7	0.06 ± 0.02
Lung	0.68 ± 0.19	2.8 ± 0.7	0.21 ± 0.07
Liver	0.28 ± 0.06	6.8 ± 1.4	0.14 ± 0.08
Spleen	0.23 ± 0.03	7.9 ± 1.5	0.09 ± 0.05
Pancreas	0.12 ± 0.02	15.7 ± 3.5	0.05 ± 0.02
Stomach (empty)	0.22 ± 0.07	8.8 ± 2.2	0.08 ± 0.02
Small intestine (empty)	0.18 ± 0.09	11.6 ± 3.9	0.07 ± 0.02
Large intestine (empty)	0.17 ± 0.03	11.0 ± 1.6	0.09 ± 0.04
Kidneys	35.3 ± 6.3	0.1 ± 0.0	16.0 ± 3.5
Adrenals	0.39 ± 0.23	5.7 ± 2.3	0.12 ± 0.04
Muscle	0.07 ± 0.01	29 ± 6	0.03 ± 0.01
Eye	1.95 ± 0.44	1.0 ± 0.2	0.09 ± 0.02
Tumor MeWo	1.86 ± 0.31		0.25 ± 0.16

References

[1] Baranyai Z, Reich D, Vágner A, et al. A shortcut to high-affinity Ga-68 and Cu-64 radiopharmaceuticals: one-pot click chemistry trimerisation on the TRAP platform. *Dalton Trans*. 2015;44:11137–11146.

[2] Reichart F, Maltsev OV, Kapp TG, Räder, AFB, Weinmüller M, Marelli UK, Notni J, Wurzer A, Beck R, Wester HJ, Steiger K, Di Maro S, Di Leva FS, Marinelli L, Nieberler M, Reuning U, Schwaiger M, Kessler H. Selective Targeting of Integrin αvβ8 by a Highly Active Cyclic Peptide. *J Med Chem*. 2019;62:2024–2037.
